# Physicochemical, Technofunctional and Antiglycaemic Properties of *Telfairia occidentalis* Leaf and Seed Powders

**DOI:** 10.1155/ijfo/5948823

**Published:** 2026-05-08

**Authors:** Mary Nkongho Tanyitiku, Igor Casimir Njombissie Petcheu

**Affiliations:** ^1^ Food and Markets Department, Natural Resources Institute, University of Greenwich, Chatham, Kent, UK, gre.ac.uk; ^2^ Medway Food Innovation Centre, Natural Resources Institute, University of Greenwich, Chatham, Kent, UK, gre.ac.uk; ^3^ Women′s Network for Biodiversity and Food Science, Yaoundé, Centre, Cameroon

**Keywords:** *α*-amylase inhibition, *α*-glucosidase inhibition, functional foods, technofunctional properties, *Telfairia occidentalis*, type 2 diabetes mellitus

## Abstract

The global prevalence of type 2 diabetes continues to rise, highlighting the need for culturally relevant, plant‐based functional ingredients capable of attenuating postprandial glycaemic responses. This study explored the physicochemical, technofunctional, antioxidant and in vitro antiglycaemic properties of *Telfairia occidentalis* leaf and seed powders, with emphasis on their application in low‐glycaemic food systems. The leaf powder (TLP) was characterised by high dietary fibre (9.42 ± 0.56 g/100 g) and polyphenol (TPC: 76.59 ± 1.62 mg GAE/g; TFC: 44.58 ± 0.77 mg QE/g), exhibiting strong antioxidant activity. In contrast, the seed powder (TSP) was rich in protein (29.52 ± 0.34 g/100 g) and lipids (19.71 ± 0.73 g/100 g), demonstrating superior oil‐holding, emulsifying and structural properties. Both fractions exhibited complementary antiglycaemic effects, including enzyme inhibition, glucose adsorption and reduced glucose diffusion, indicating multiple mechanisms of action. TSP exhibited moderate *α*‐amylase (IC_50_: 2580 ± 0.04 *μ*g/mL) and *α*‐glucosidase inhibition (IC_50_: 2230 ± 0.00 *μ*g/mL), although lower than TLP (*α*‐amylase IC_50_: 1530 ± 0.08 *μ*g/mL; *α*‐glucosidase inhibition IC_50_: 840 ± 0.02 *μ*g/mL) but was higher than acarbose (IC_50_: 530 ± 0.05 and 330 ± 0.02 *μ*g/mL, respectively). Scanning electron micrographs and Fourier‐transform infrared (FTIR) spectra revealed distinct microstructural and molecular differences between TLP and TSP, highlighting a fibre‐rich porous matrix in the leaf and a protein–lipid‐dominated structure in the seed. Functionally, TLP contributes fibre‐ and polyphenol‐mediated glucose regulation and antioxidant protection, whereas TSP provides protein–lipid‐driven functional performance and modulation of glucose release and transport. These findings suggest that integrating TLP and TSP into composite formulations represents a promising strategy for developing low‐glycaemic, antioxidant‐rich foods with potential relevance for type 2 diabetes management.

## 1. Introduction

Globally, an estimated 537 million adults lived with diabetes in 2021, and this number is projected to rise to 783 million by 2045 [1]. Approximately 90% of these cases are type 2 diabetes mellitus (T2DM), representing one of the most pressing public health challenges of the 21st century [1, 2]. Blood glucose is the body′s main source of energy and is derived from the food we eat. However, when the pancreas is unable to produce sufficient insulin, or when the body′s cells become resistant to insulin, glucose uptake is impaired, resulting in elevated levels of glucose in the bloodstream. Postprandial hyperglycaemia, an excessive rise in blood glucose following meals, has emerged as an independent predictor of microvascular and macrovascular complications, including cardiovascular disease, oxidative stress and endothelial dysfunction [3–5]. Although conventional therapies such as *α*‐glucosidase inhibitors and insulin sensitizers can mitigate hyperglycaemia, they are often limited by adverse effects, poor long‐term adherence and high costs, especially in resource‐limited settings [5, 6]. As such, these limitations have increased interest in plant‐based functional foods, which deliver bioactive compounds that support metabolic health [6–8].

Functional foods provide health benefits beyond basic nutrition and commonly contain plant‐derived components such as dietary fibres, phenolic compounds and bioactive proteins that modulate key physiological processes [2, 5]. This is particularly evident in many medicinal and edible plants, which naturally accumulate bioactive molecules with demonstrated metabolic regulatory effects [5]. These antioxidant‐rich plants or plant extracts offer a notable advantage, as they are generally associated with fewer adverse effects than conventional antidiabetic drugs, while still providing bioactive compounds capable of modulating glucose‐related metabolic pathways [9]. More recently, plant‐derived components from underutilised crops have attracted growing interest owing to their rich micronutrient profiles and their potential to address both metabolic disorders and food‐security challenges [10–12].

*Telfairia occidentalis* (fluted pumpkin) is a perennial cucurbit that is cultivated primarily in West Africa, where the leaves are widely consumed in soups and stews, and the seeds are eaten roasted, boiled or processed into pastes [10, 13]. Although global production figures are not formally documented, the crop holds considerable regional dietary and cultural importance with expanding cultivation beyond West Africa [7, 12, 14]. Beyond their culinary use, both the leaves and seeds are traditionally valued for their medicinal properties, including haematopoietic, anti‐inflammatory and antidiabetic effects [10–12, 15]. Despite this prominence, *T. occidentalis* remains, to our knowledge, underexplored as a functional food ingredient. Previous studies [7, 11, 13, 15, 16] have focused largely on the nutritional, antimicrobial and pharmacological properties of its leaf and seed extracts, while comparatively little attention has been given to their technofunctional attributes. These include water‐ and oil‐holding capacities, emulsification, foaming, gelation and related structural behaviours that underpin functionality in sustainable food systems. In addition, the ways in which their intrinsic composition, such as proteins, dietary fibres, phenolics and natural lipids, could interact to influence structure formation, enzyme accessibility and glucose‐regulatory mechanisms remain insufficiently characterised. Addressing these gaps is timely given the rising global prevalence of type 2 diabetes (T2D) and the increasing demand for affordable, culturally relevant dietary strategies to attenuate postprandial hyperglycaemia.

Consequently, this study is aimed at exploring and comparatively characterising the physicochemical, technofunctional, antioxidant and in vitro antiglycaemic properties of *T. occidentalis* leaf and seed powders. By systematically linking bioactivity with technofunctional performance and microstructural characteristics, this work provides a foundational assessment of their potential for incorporation into functional food formulations designed to moderate glucose release and support metabolic health.

## 2. Materials and Methods

### 2.1. Materials

**Figure 1:**
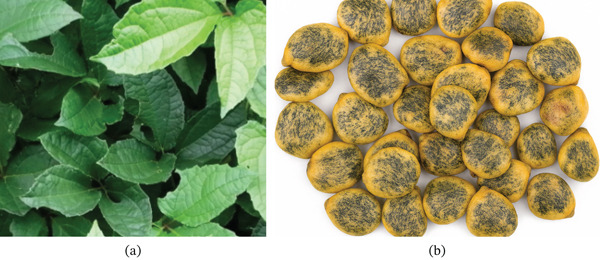
(a) Fresh leaves and (b) pods of *T. occidentalis.*

Fresh mature leaves and pods of *T. occidentalis* (Figure 1) were collected from a local farm in Mokolo, Yaoundé, Cameroon, 100 days after planting. The leaves were manually sorted, destalked and thoroughly washed with deionised water to remove surface impurities. After draining, they were lightly crushed, frozen at −18°C for 24 h and then freeze‐dried (−50°C, 0.1 mbar) for 48 h. The pods were sun‐dried for 5 days at 25°C ± 5°C, manually cracked open and dehulled by gentle abrasion. The seeds were then rinsed with deionised water and freeze‐dried for 36 h. Both leaves and seeds were separately ground into powder and sieved (< 250 *μ*m) to obtain *T. occidentalis* leaf powder (TLP) and seed powder (TSP). TLP and TSP were stored in airtight polyethylene bags until analysis. All chemicals and reagents were of analytical grade.

### 2.2. Proximate Analysis

The proximate composition of TLP and TSP was determined by quantifying moisture, ash, crude fat, protein, starch and dietary fibre contents. Moisture and lipid contents were analysed according to AOAC Official Method 2008.06. Ash content was determined gravimetrically by incinerating approximately 2.5 g of each sample in a muffle furnace at 550°C for 24 h. Total nitrogen was measured using the Dumas combustion method (AOAC Official Method 992.15), and protein content was calculated using a conversion factor of 6.25. Dietary fibre fractions were determined using an enzymatic‐gravimetric method (AOAC Method 991.43), with insoluble dietary fibre (IDF) and soluble dietary fibre (SDF) quantified separately; total dietary fibre (TDF) was expressed as the sum of IDF and SDF. Total starch content was assessed using a commercial Total Starch Assay Kit (Merck Life Science, Sigma‐Aldrich, Gillingham, United Kingdom).

### 2.3. Protein Solubility

Protein solubility was determined as described by Kaur and Singh [17], with slight modifications. Suspensions of TSP or TLP (1%, w/v) were prepared in deionised water, and the pH was adjusted to between 3 and 9 using 0.1 M HCl or 0.1 M NaOH. Samples were incubated at 40°C for 60 min and then centrifuged at 3000 × g for 20 min. Protein solubility (%) was calculated as the proportion of soluble protein in the supernatant relative to the total protein content of the original sample.

### 2.4. Water‐Holding Capacity (WHC) and Oil‐Holding Capacity (OHC)

WHC and OHC were determined according to Kaur and Singh [17]. TLP or TSP samples (0.25 g) were combined with 5 mL of deionised water (for WHC) or 5 mL of rapeseed oil (for OHC) and gently agitated at 25^°^C ± 2^°^C for 30 min. The mixtures were centrifuged at 3000 × g for 10 min, and the volume of the recovered supernatant was recorded. WHC or OHC were calculated as the difference between the initial liquid volume added and the supernatant volume recovered, multiplied by the density of the liquid (1.00 g/mL for water or 0.91 g/mL for the oil) and expressed per gram of sample.

### 2.5. Emulsifying Properties

Emulsifying activity index (EAI) and emulsion stability index (ESI) were determined following Kaur and Singh [17]. TLP or TSP samples (0.5% w/v) were homogenised with rapeseed oil (3:1 w/v) at 10,000 rpm and 25°C for 2 min. An aliquot of the emulsion was then diluted (1:100) with 0.1% sodium dodecyl sulfate. The absorbance at 500 nm was recorded (A0) and then after 10 min (A10). EAI (m^2^/g) and ESI (min) were calculated using the following equations:

EAI m2g=22.3030××A×DC×ɸ×L×10,000

ESI min=A0A010−A×10

where *C* is the protein concentration (g/mL), *D* is the dilution factor, *ϕ* is the oil volume fraction (0.25) and *L* is the path length of the cuvette (cm).

### 2.6. Foaming Properties

Foaming capacity (FC) and foaming stability (FS) were assessed according to Kaur and Singh [17]. A 1% (w/v) suspension of TLP or TSP was whipped at 12,000 rpm for 2 min using a hand‐held homogeniser in a graduated cylinder. The initial foam volume was recorded immediately and then after 30 min. FC was calculated as the percentage increase in volume immediately after whipping relative to the initial liquid volume, whereas FS was expressed as the percentage of the initial foam volume that remained after 30 min.

### 2.7. Least Gelation Concentration (LGC)

LGC was determined as described by Kaur and Singh [17]. TLP or TSP suspensions (3% w/v) were prepared in deionised water and heated in a water bath at 90°C for 1 h. The samples were then rapidly cooled under running water and refrigerated at 4°C for 2 h. LGC was defined as the lowest concentration at which the gel remained stationary upon inversion of the tube.

### 2.8. Flowability

The flowability of TLP and TSP was assessed according to Awari et al. [18]. For bulk density, the powders were gently filled into a 50‐mL graduated cylinder to the marked volume. The settled volume was recorded when the cylinder was dropped from a height of 1 in at 2‐s intervals. Tapped density was obtained after 500 taps, once the volume had stabilised. Carr index (CI) and Hausner ratio (HR) were calculated from bulk and tapped density values, where CI quantifies powder compressibility and HR is the ratio of tapped to bulk density, serving as an indicator of cohesiveness and flowability. According to established criteria [18, 19], CI values of 5%–15% and HR values of 1.0–1.1 indicate excellent flow, whereas CI > 25% or HR > 1.4 reflect poor flow characteristics.

The angle of repose (*θ*) was determined by allowing the powder to flow freely through a funnel positioned 6 cm above a flat surface, forming a conical heap [18]. Angles near 30° indicate good flowability, whereas values approaching 40° suggest reduced flowability.

### 2.9. Colour

Colour parameters were recorded using a Minolta Chroma Meter (CR‐400, Konica Minolta, Japan) calibrated against a standard white tile. Measurements were expressed in the CIE Lab colour system, where *L* denotes lightness (0 = black; 100 = white), a* represents the green (−) to red (+) axis, and b* represents the blue (−) to yellow (+) axis. Chroma (C*) and hue angle (h°) were calculated from the L*, a* and b* coordinates. Total colour difference (*Δ*E*) was determined relative to the reference white standard.

### 2.10. Total Phenolic Content (TPC)

TLP and TSP extracts were prepared using an aqueous maceration procedure adapted from Oluwagunwa et al. [8]. Aqueous extraction was selected because (1) it produced a higher TPC (12.2%) and stronger free‐radical–scavenging activity (92%) compared with ethanolic extraction (total phenolics: 5.5%; scavenging activity: 25%) [11], and (2) to better reflect likely food and gastrointestinal conditions. Briefly, 1 g of each powder was mixed with 20 mL of deionised water in a sterile glass tube. The mixture was subjected to ultrasonic treatment at 65°C for 30 min, followed by maceration at 350 rpm and 25°C for 4 h. The suspension was then centrifuged at 4000 × g for 10 min, and the resulting supernatant was collected and stored at 4°C until further analysis.

TPC was quantified as described by Awari et al. [18], with minor modifications. An aliquot (0.2 mL) of each extract was mixed with 1.6 mL of deionised water, followed by 0.1 mL of Folin‐Ciocalteu reagent and 0.3 mL of sodium carbonate solution (20%, w/w). The mixture was vortexed and incubated at 25 ± 2°C in the dark for 60 min. The final volume was adjusted to 4 mL with deionised water, and the absorbance was measured at 750 nm. A gallic acid calibration curve (0.01–0.1 mg/mL) was used, and results were expressed as mg GAE/g of extract.

### 2.11. Total Flavonoid Content (TFC)

TFC was determined using the aluminium chloride colorimetric method [18]. Briefly, appropriate volumes of each extract were reacted with aluminium chloride reagent and potassium acetate in an aqueous medium. The mixture was incubated at 25^°^C ± 2^°^C for 30 min to allow formation of the flavonoid–aluminium complex, characterised by a yellow coloration. Absorbance was then measured at 415 nm. Quercetin served as the reference standard, and the results were expressed as mg QE/g of extract.

### 2.12. DPPH·Radical Scavenging Activity

Free‐radical scavenging capacity was evaluated using the DPPH assay according to Thaipong et al. [20]. A 0.2‐mL aliquot of each extract was mixed with 2.8 mL of freshly prepared 0.1‐mM DPPH solution in methanol. The reaction mixture was vortexed and incubated in the dark at room temperature for 30 min. The decrease in absorbance was measured at 517 nm using a UV‐Vis spectrophotometer. Antioxidant capacity was calculated from a Trolox calibration curve (0.05–2 mM) and expressed as *μ*mol TE per mL of extract.

### 2.13. ABTS•^+^ Radical Cation Scavenging Activity

The ABTS•^+^ assay was conducted following the method of Thaipong et al. [20], with slight modifications. The ABTS radical cation was generated by reacting 7‐mM ABTS with 2.45‐mM potassium persulfate and allowing the mixture to stand in the dark at 4°C for 16 h. Prior to analysis, the radical solution was diluted with 50‐mM phosphate‐buffered saline (pH 7.4) to obtain an absorbance of 0.70 ± 0.02 at 734 nm. Subsequently, 10 *μ*L of each extract (1.0 mg/mL final concentration) was added to 990 *μ*L of the diluted ABTS solution. After incubation in the dark at room temperature for 6 min, absorbance was recorded at 734 nm.

### 2.14. Ferric Reducing Antioxidant Power (FRAP)

FRAP was determined according to the method of Thaipong et al. [20], with minor modifications. An aliquot of each extract (140 *μ*L; final concentration 2.27 mg/mL) was mixed with 140 *μ*L of phosphate buffer (200 mM, pH 6.6) and 140 *μ*L of potassium hexacyanoferrate (III) solution (10 mg/mL). The reaction mixture was incubated at 50°C for 20 min. The reaction was terminated by adding 200 *μ*L of trichloroacetic acid (100 mg/mL), followed by centrifugation at 2000 × g for 10 min. A portion of the resulting supernatant (400 *μ*L) was then combined with 400 *μ*L of distilled water and 80 *μ*L of ferric chloride solution (1 mg/mL). After standing in the dark for 10 min, absorbance was measured at 700 nm.

### 2.15. *α*‐Amylase Inhibitory Activity

Amylase inhibitory activity was measured as described by Patil et al. [21] with minor modifications. The substrate solution was prepared by dissolving 500 mg of soluble starch in 25 mL of 0.4‐M NaOH, heating at 100°C for 5 min, adjusting the pH to 7.0 with HCl and diluting to a final volume of 100 mL with distilled water. Plant extract or acarbose standard solutions of varying concentrations (0.5–2.5 mg/mL) were prepared in acetate buffer (pH 6.5). The enzyme inhibition assays were carried out at low sample concentrations (0.5–2.5 mg/mL) to ensure that the observed effects primarily reflected intrinsic polyphenol and protein–enzyme interactions rather than nonspecific viscosity or matrix‐driven physical interference.

In each microplate well, 20 *μ*L of plant extract, 40 *μ*L of substrate solution and 20 *μ*L of *α*‐amylase solution (50 *μ*g/mL) were combined and incubated at 25°C for 15 min. The reaction was terminated by adding 80 *μ*L of 0.1‐M HCl and 200 *μ*L of 1‐mM iodine solution to form a starch–iodine complex. Absorbance was measured at 650 nm. The IC_50_ (mg/mL) was defined as the concentration required for an inhibitor to reduce 50% of the enzyme activity [9]. It was determined from the regression analysis of percentage inhibition values plotted against sample concentrations.

### 2.16. *α*‐Glucosidase Inhibitory Activity

The *α*‐glucosidase inhibitory activity of TSP and TLP was determined following the method described by Ruth et al. [10]. Briefly, 250 *μ*L of plant extract or acarbose standard solution (0.5–2.5 mg/mL) was incubated with 100 *μ*L of *α*‐glucosidase (2 IU) at 37°C for 15 min. Subsequently, 250 *μ*L of 4‐nitrophenyl *β*‐D‐glucopyranoside solution (5 mM) was added, and the mixture was further incubated at 37°C. Absorbance readings were taken at 405 nm, and inhibitory activity was expressed as % inhibition. The IC_50_ value (mg/mL) was determined from the regression analysis of percentage inhibition values plotted against sample concentrations.

### 2.17. Pancreatic Lipase Inhibitory Activity

Lipase inhibitory activity was determined according to Patil et al. [21] with slight modifications. The substrate solution was prepared by dissolving lecithin (10 mg), sodium cholate (5 mg) and glycerol trioleate (80 mg) in 9 mL of 0.1‐M TES buffer (pH 7.0). Extracts or orlistat standard solution of varying concentrations (0.5–2.5 mg/mL) were prepared in the same buffer. In each microplate well, 20 *μ*L of sample and 20 *μ*L of substrate solution were combined, followed by the addition of 10 *μ*L of lipase solution (20 *μ*g/mL). The mixture was incubated at 37°C for 30 min, and absorbance was measured at 550 nm using a microplate reader. Lipase inhibitory activity (%) was calculated as the percentage decrease in absorbance of the sample relative to the control. IC_50_ values were visually approximated from dose‐response curves (0.5–2.5 mg/mL) based on the concentration required to achieve 50% inhibition.

### 2.18. Glucose Adsorption Capacity

Glucose adsorption capacity was determined following the method described by Shirkhan et al. [5] with minor modifications. In brief, 0.1 g of TLP or TSP was added to 20 mL of glucose solutions at concentrations of 20, 40, 50, 60 and 100 mM. The mixture was shaken at 37°C for 6 h and then centrifuged at 4000 × g for 15 min. A 1.0‐mL aliquot of the supernatant was mixed with 3 mL of DNS reagent and heated in a water bath at 100°C for 5 min to facilitate colour development. Glucose concentration in the supernatant was subsequently measured using a glucose oxidase kit. Glucose adsorption capacity (mmol/g) was calculated as the difference between initial and final glucose concentrations, expressed per gram of sample. The sample without TLP or TSP served as the control.

### 2.19. Glucose Diffusion

The effect of TLP and TSP on glucose transport was evaluated using a dialysis membrane–based in vitro glucose diffusion model, as described by Shirkhan et al. [5]. Briefly, 0.375 g of TLP or TSP was dispersed in 188.75 mL of glucose solution (10 mM) and homogenised, followed by incubation at 37°C for 10 min to allow glucose–matrix interactions. Aliquots (10 mL) of each mixture were transferred into prehydrated dialysis tubing (12 kDa), sealed and immersed in 100 mL of deionised water maintained at 37°C with continuous agitation (100 rpm). At 0, 30, 60, 90 and 120 min, 2 mL of the external dialysate was withdrawn, and glucose concentration was determined using a glucose oxidase–peroxidase kit. Glucose diffusion was expressed as the percentage of glucose diffused into the dialysate relative to the initial glucose concentration. A glucose solution without sample served as the control.

### 2.20. Scanning Electron Microscopy

The surface morphology of TLP and TSP powders was characterised using a field‐emission scanning electron microscope (ZEISS GeminiSEM 500, Carl Zeiss, Oberkochen, Germany). Samples were sputter‐coated with a thin gold‐palladium layer under vacuum for approximately 60 s, and micrographs were acquired at accelerating voltages between 5 and 15 kV across magnifications ranging from 3000× to 10,000×.

### 2.21. Fourier Transform Infrared (FTIR) Spectroscopy

The molecular and structural characteristics of TLP and TSP were investigated using a FTIR spectrometer (PerkinElmer Spectrum Two, L1600235, Beaconsfield, Bucks, United Kingdom). Spectral acquisition and processing were performed using Spectrum IR software (Version 10.6.2). Infrared spectra were recorded over the midinfrared region (4000–400 cm^−1^), with 32 scans averaged per sample at a resolution of 4 cm^−1^. To further examine protein secondary structure, the Amide I region (1700–1600 cm^−1^) was additionally acquired at a higher resolution (2 cm^−1^) with 64 scans to improve spectral definition. Preliminary deconvolution and Gaussian peak fitting of the Amide I band were performed to explore potential contributions from *α*‐helix, *β*‐sheet, *β*‐turn and random coil structures. However, due to substantial band overlap and the heterogeneous nature of the samples, quantitative assignment of secondary structure components was limited. Consequently, interpretation of this region was based primarily on qualitative assessment of band position, intensity and broadening. Characteristic absorption bands corresponding to hydroxyl (O–H), carbonyl (C=O), ether (C–O–C) and amine (N–H) functional groups were identified and compared between leaf and seed powders. These spectral features were interpreted in relation to the compositional constituents of the samples, including polyphenols, proteins, carbohydrates and lipids, and were used to support discussion of potential intermolecular interactions influencing technofunctional and antiglycaemic properties.

### 2.22. Statistical Analysis

All measurements were conducted in triplicate, consistent with standard practice in exploratory in vitro studies; however, no formal power analysis was performed, which should be considered in future validation studies. Statistical analyses were conducted using IBM SPSS Statistics Version 31.0.0.0 (IBM Corp., Armonk, New York, United States), and results are presented as mean ± standard deviation. Differences among means were evaluated by one‐way analysis of variance (ANOVA), followed by Tukey′s honestly significant difference test for pairwise comparisons when significant effects were detected. Statistical significance was accepted at *p* < 0.05. Different superscript letters within the same row or figure indicate values that differ significantly.

## 3. Results and Discussion

### 3.1. Proximate Composition

The proximate composition of TLP and TSP is presented in Table 1. TLP exhibited a notably high TDF content (9.42 ± 0.56 g/100 g), comprising both insoluble and soluble fractions, along with appreciable levels of protein (10.22 ± 0.55 g/100 g), starch (9.87 ± 0.68 g/100 g) and ash (7.60 ± 0.48 g/100 g), underscoring its potential as a nutrient‐dense functional ingredient. Idris [13] similarly reported substantial fibre (20.17 ± 0.12 g/100 g), protein (8.72 ± 0.03 g/100 g) and ash (17.2 ± 0.02 g/100 g) contents in fluted pumpkin leaves collected in Niger State, Nigeria. Fasuyi [22] documented even higher protein levels (35.1 ± 1.7 g/100 g), surpassing those of *Amaranthus cruentus* (23.0 ± 1.3 g/100 g) and *Talinum triangulare* (19.9 ± 1.8 g/100 g). The protein content of TLP in the present study is comparable with values reported for blackberry leaves (24.34%) and moringa leaf flour (6.72%) [23]. In contrast, Bayang et al. [24] reported higher protein concentrations in other African leafy vegetables, including *Corchorus olitorius* (25.67 g/100 g), *Cucumis melo* (28.46 g/100 g), *Momordica charantia* (30.71 g/100 g), *Cleome gynandra* (34.87 g/100 g) and *Hibiscus sabdariffa* (39.10 g/100 g).

**Table 1:** Proximate composition of *T. occidentalis* leaf and seed powders.

Macronutrients	TLP	TSP
Moisture (g/100 g)	5.45 ± 0.22^a^	4.32 ± 0.80^a^
Ash (g/100 g)	7.60 ± 0.48^b^	5.33 ± 0.92^a^
Protein (g/100 g)	10.22 ± 0.55^b^	29.52 ± 0.34^a^
Fats (g/100 g)	3.24 ± 0.20^b^	19.71 ± 0.73^a^
Starch (g/100 g)	9.87 ± 0.68^b^	12.56 ± 0.61^a^
Soluble fibre (g/100 g)	2.74 ± 0.32^a^	1.97 ± 0.59^a^
Insoluble fibre (g/100 g)	6.50 ± 0.08^b^	4.29 ± 0.73^a^
Total dietary fibre (g/100 g)	9.42 ± 0.56^b^	5.78 ± 0.95^a^

*Note:* Results are expressed as mean ± standard deviation (*n* = 3), and means with different superscripts in the same row are significantly (*p* < 0.05) different.

Abbreviations: TLP, *T. occidentalis* leaf powder; TSP, *T. occidentalis* seed powder.

The elevated dietary fibre content of TLP is particularly relevant for glycaemic regulation, as both soluble and insoluble fibre fractions can attenuate postprandial glucose responses by increasing intestinal viscosity, delaying gastric emptying and reducing glucose diffusion across the intestinal epithelium [8, 25]. Furthermore, leafy vegetables rich in fibre and polyphenols have been associated with lower glycaemic index values and improved insulin sensitivity [12, 25], supporting the potential application of TLP as a functional ingredient for diabetes‐oriented food formulations.

TSP, in contrast, was characterised by its markedly higher protein content (29.52 ± 0.34 g/100 g) and substantial fat levels (19.71 ± 0.73 g/100 g), consistent with its role as an energy‐dense storage tissue. Previous studies [26, 10, 12] on *T. occidentalis* seeds have reported similar protein values (21.46%–30.28%) and fat contents ranging from 28.13% to 37.87%. These values are comparable with other tropical legumes and oilseeds commonly incorporated into food products, including groundnut (10.95 ± 0.41–27.00 ± 0.08 g/100 g) [26], watermelon seeds (6.1*%* ± 0.06*%*–49.70*%* ± 0.61*%*) [27], pumpkin seeds (37.67 ± 0.20 g/100 g) [4], *Amaranthus viridis* seeds (8.98*%* ± 0.20*%*–18.07*%* ± 0.47*%*) [28], and okra seeds (16.80%–17.40%) [25]. The relatively high fat content of TSP is nutritionally and technologically important, as dietary lipids can slow gastric emptying and starch hydrolysis, thereby reducing the rate of postprandial glucose appearance [4].

From a food‐processing perspective, the coexistence of proteins and lipids in *T. occidentalis* seed powder may enhance emulsifying capacity, oil‐holding ability and mouthfeel, making TSP a suitable ingredient for incorporation into soups, sauces, spreads, and baked goods. Notably, full‐fat seed powder was used to preserve the native protein–lipid matrix and bioactive lipophilic constituents, thereby allowing evaluation of the ingredient in its minimally processed, food‐relevant form and capturing potential synergistic effects of proteins and lipids on technofunctional and antiglycaemic properties. Although TLP contained lower protein and lipid contents than TSP, it exhibited significantly higher levels of dietary fibre and ash, indicating a complementary nutritional profile. Taken together, these compositional differences suggest the potential for synergistic health benefits, a fibre‐ and polyphenol‐mediated glycaemic modulation from the leaf powder combined with protein‐ and lipid‐driven functional and metabolic contributions from the seed powder. Such synergy is particularly valuable for the development of low‐glycaemic, nutrient‐dense functional foods targeted at diabetes management [12].

### 3.2. Total Phenolic and Flavonoid Contents

In Table 2, TLP exhibited significantly (*p* < 0.05) higher TPC (76.59 mg GAE/g) and TFC (44.58 mg QE/g) compared with TSP, suggesting the leaves could reserve higher polyphenolic compounds in the plant. Similar high phenolic and flavonoid contents have been reported for fluted pumpkin leaves collected from Lome, Togo, with TPC values of 173.7 ± 0.05 mg GAE/g and TFC of 139.62 ± 0.06 mg QE/g [29]. The dominance of polyphenols in TLP is of major functional significance. Polyphenolic compounds, particularly flavonoids, have been widely reported to inhibit key carbohydrate‐digesting enzymes, including *α*‐amylase and *α*‐glucosidase, thereby reducing the rate of starch hydrolysis and glucose release in the gastrointestinal tract [4, 6]. The high TPC and TFC observed in TLP in this study therefore provide a biochemical basis for its strong enzyme inhibitory and antiglycaemic activities observed in this research.

**Table 2:** TPC, TFC and antioxidant properties of *T. occidentalis* leaf and seed powders.

Properties	TLP	TSP
TPC (mg GAE/g)	76.59 ± 1.62^a^	24.35 ± 2.34^b^
TFC (mg QE/g)	44.58 ± 0.77^a^	13.89 ± 0.94^b^
DPPH·scavenging (%)	82.67 ± 0.45^a^	38.71 ± 0.60^b^
ABTS·^₊^ (*μ*mol TE/g)	4.87 ± 0.54^a^	1.94 ± 0.68^b^
FRAP (*μ*mol Fe^2+^/g)	3.56 ± 0.83^a^	1.56 ± 0.83^b^

*Note:* Results are expressed as mean ± standard deviation (*n* = 3), and means with different superscripts in the same row are significantly (*p* < 0.05) different.

In contrast, TSP contained moderate levels of phenolic and flavonoid compounds (24.35 mg GAE/g and 13.89 mg QE/g, respectively), indicating appreciable quantities of phenolics despite their high lipid content. Importantly, the presence of polyphenols in TSP, together with its high protein and lipid contents, suggests a synergistic mode of action in glycaemic regulation [13]. While the phenolic fraction may contribute directly to enzyme inhibition and antioxidant effects, the protein–lipid matrix can slow gastric emptying and starch digestion, further reducing the rate of glucose appearance in the bloodstream [4, 10]. This combination of moderate polyphenol activity with macronutrient‐mediated modulation of digestion distinguishes TSP from the leaf powder while supporting its complementary role in functional food formulations. Because the present study employed water as the extraction solvent to better reflect likely food and gastrointestinal conditions, the phenolic and antioxidant values reported may not be directly comparable with studies using ethanolic or methanolic extraction, which typically recover a broader range of both polar and moderately nonpolar phenolic compounds.

### 3.3. Antioxidant Activity

The antioxidant capacities of TLP and TSP are presented in Table 2. TLP exhibited significantly (*p* < 0.05) higher antioxidant activity across all three assays, with DPPH· scavenging values of 82.67*%* ± 0.45*%*, ABTS·^₊^ values of 4.87 ± 0.54 *μ*mol Trolox equivalents/g and FRAP values of 3.56 ± 0.83 *μ*mol Fe^2+^ equivalents/g. These results confirm that the leaf is the dominant antioxidant component of the fluted pumpkin plant. Comparable high antioxidant activities have been reported for fluted pumpkin leaves [11, 29] and other African leafy vegetables [24, 30], which are rich in polyphenols, flavonoids and chlorophyll‐associated compounds.

The strong antioxidant capacity of TLP is consistent with its high total phenolic and flavonoid contents (Table 2). Polyphenols act as electron or hydrogen donors, neutralizing reactive oxygen species and preventing oxidative damage to biomolecules such as lipids, proteins and nucleic acids. In the context of diabetes, oxidative stress plays a central role in pancreatic *β*‐cell dysfunction and insulin resistance [1, 3]. Therefore, the high radical scavenging and reducing power observed for TLP suggests that it may help protect pancreatic *β*‐cells from oxidative injury and mitigate the progression of hyperglycaemia‐induced tissue damage [6, 10].

In contrast, TSP exhibited moderate but significant antioxidant activity (DPPH: 38.71*%* ± 0.60*%*; ABTS·^₊^: 1.94 ± 0.68 *μ*mol TE/g; FRAP: 1.56 ± 0.83 *μ*mol Fe^2+^/g). Its antioxidant activity could be due to the presence of phenolic compounds as well as protein‐associated peptides, which can quench free radicals and chelate pro‐oxidant metals [10]. These antioxidant mechanisms are particularly important for preventing lipid oxidation, thereby improving the oxidative stability and shelf life of food products formulated with TSP. Overall, whereas TLP could provide potent aqueous‐phase antioxidant protection that is relevant to cellular oxidative stress and glycaemic control [6, 12], TSP could contribute lipid‐phase antioxidant activity that enhances food stability and also modulate metabolic oxidative pathways [10].

### 3.4. Technofunctional Properties

#### 3.4.1. Protein Solubility

**Figure 2:**
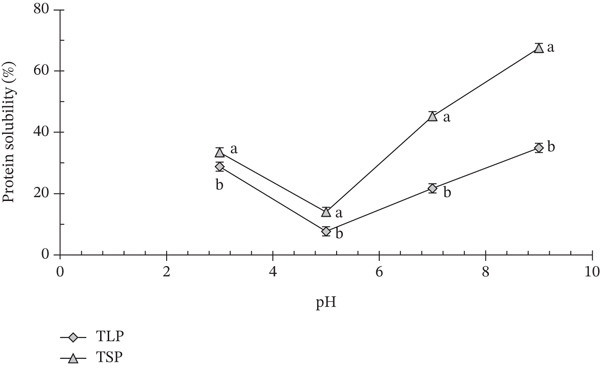
Protein solubility trend of *T. occidentalis* leaf and seed powders. Values are mean ± SD (*n* = 3). Different superscript letters at the same time point indicate significant differences (*p* < 0.05) as determined by one‐way ANOVA followed by Tukey′s HSD test.

The protein solubility profiles of TLP and TSP exhibited a typical U‐shaped trend across the evaluated pH range (3–9), with significant differences (*p* < 0.05) observed between the samples, particularly around their isoelectric region (Figure 2). Both powders showed minimum solubility at pH 5, indicating proximity to their isoelectric point, where net protein charge is minimal and protein–protein aggregation is maximised. At this pH, solubility decreased to approximately 6%–8% for TLP and 12%–15% for TSP. Solubility increased progressively under alkaline conditions, reflecting enhanced electrostatic repulsion and improved protein dispersion. At pH 9, TSP exhibited significantly (*p* < 0.05) higher protein solubility (67.45%) than TLP (34.89%), indicating greater protein extractability and functional potential under alkaline environments.

The consistently higher solubility of TSP across most pH values suggests superior protein dispersibility, likely attributable to its higher protein content and the presence of more soluble globulin‐type storage proteins typical of oilseeds. Comparable solubility ranges (55%–70%) have been reported for oilseed flours and pumpkin seed proteins, which are noted for their good dispersibility and emulsifying capabilities [3, 4]. In contrast, the lower solubility observed for TLP may reflect the strong association of leaf proteins with cell‐wall polysaccharides, structural fibres and polyphenols, which reduce hydration, limit protein unfolding and restrict extractability [26]. Polyphenol–protein interactions, mediated through hydrogen bonding and hydrophobic forces, are particularly common in leafy vegetables and can suppress solubility while simultaneously enhancing antioxidant and enzyme‐inhibitory activities [28]. Similar moderate solubility behaviour has been reported for polyphenol‐rich seed flours such as *Amaranthus* spp. [28] and watermelon seeds [27].

Overall, whereas TSP contributes highly soluble, surface‐active proteins suited to emulsified and aerated food systems, TLP offers structurally bound proteins associated with fibre‐ and polyphenol‐mediated glycaemic modulation. This complementary behaviour provides desirable processing and health‐related attributes for developing functional foods with metabolic benefits [10].

#### 3.4.2. WHC and OHC

The WHC and OHC of TLP and TSP are presented in Table 3. TLP exhibited significantly higher WHC (4.71 ± 0.42 g/g) than TSP, reflecting its high dietary fibre and polysaccharide content. Similar high WHC values have been reported for fluted pumpkin leaves and other African leafy vegetables, confirming their suitability for thickening and moisture retention in food systems [3, 31]. High WHC values are typical of fibrous leafy vegetable powders, which contain abundant hydroxyl groups capable of binding water through hydrogen bonding [1, 2]. The high WHC of TLP is also nutritionally relevant as dietary fibres with high hydration capacity increase digesta viscosity and entrap glucose, thereby slowing glucose diffusion and absorption in the intestine [4]. This supports the observed glucose‐modulating and enzyme inhibitory activities of TLP, reinforcing its potential as a low‐glycaemic functional ingredient.

**Table 3:** Functional properties of *T. occidentalis* leaf and seed powders.

Parameters	TLP	TSP
WHC (g/g)	4.71 ± 0.42^a^	3.24 ± 0.40^b^
OHC (g/g)	1.45 ± 0.55^b^	4.23 ± 0.21^a^
FC (%)	23.63 ± 0.08^a^	18.45 ± 0.02^b^
FS (%)	11.89 ± 0.05^a^	6.57 ± 0.11^b^
EAI (m^2^/g)	19.43 ± 0.62^b^	44.35 ± 0.44^a^
ESI (min)	24.65 ± 0.88^b^	65.41 ± 0.23^a^
LGC (%)	8.90 ± 0.42^b^	11.71 ± 0.51^a^

*Note:* Results are expressed as mean ± standard deviation (*n* = 3), and means with different superscripts in the same row are significantly (*p* < 0.05) different.

Abbreviations: TLP, *T. occidentalis* leaf powder; TSP, *T. occidentalis* seed powder.

In contrast, TSP showed a moderate WHC (3.24 ± 0.4 g/g) but a significantly (*p* < 0.05) higher OHC (4.23 ± 0.21 g/g), which can be attributed to its high lipid and protein contents. Comparable high OHC values have been reported for oil‐rich seed flours such as pumpkin seed [4], sesame [32] and groundnut [26], which are widely used in flavour‐rich and emulsified food products. OHC in flours is largely governed by nonpolar amino acid side chains and lipid–protein interactions, which enhance the binding of hydrophobic compounds [26, 28]. As such, the high OHC of TSP makes it particularly suitable for applications such as soups, spreads, sauces and bakery fillings, where oil retention is critical for mouthfeel and flavour delivery.

#### 3.4.3. Emulsifying Capacity and Stability

The emulsifying properties of TLP and TSP are presented in Table 3.

TSP exhibited significantly (*p* < 0.05) higher EAI (44.35 ± 0.44 m^2^/g) and ESI (65.41 ± 0.23 min) compared with TLP, reflecting its superior ability to form and stabilise oil‐in‐water emulsions. Here, TLP displayed lower EAI (19.43 ± 0.62 m^2^/g) and ESI (24.65 ± 0.88 min), consistent with its low protein and lipid contents. Although the leaf powder contains polysaccharides and polyphenols that may provide some viscosity‐mediated stabilization, the absence of a substantial protein–lipid interface limits its emulsifying performance [18, 26]. Comparatively, Ochoa‐Rivas et al. [33] observed lower values of fat absorption index (2.32 mL/g) but higher emulsifying activity (68.45%) in peanut seed flour. The high EAI and ESI of TSP are attributable to its protein–lipid matrix, where surface‐active proteins adsorb at the oil–water interface while lipids contribute to interfacial cohesion and emulsion stability [26]. In addition, the higher protein solubility of TSP provides a strong mechanistic basis for its superior EAI and ESI, as soluble proteins can rapidly adsorb at oil–water interfaces, unfold and form viscoelastic interfacial films that stabilise emulsions. This explains the excellent emulsifying and foaming performance observed for TSP despite its high lipid content. Moreover, soluble seed proteins are more accessible to digestive enzymes, allowing controlled hydrolysis while still forming protein–lipid matrices that slow glucose and lipid diffusion, thereby contributing to the observed antiglycaemic and antilipase effects [6, 27].

The high emulsifying activity and stability of TSP have important implications for functional food formulation. The ability to stabilise emulsions makes TSP suitable for soups, sauces, beverages and bakery fillings, where oil retention and interfacial stability are critical for texture and mouthfeel [13]. In composite formulations, TLP and TSP could provide bioactive compounds, fibre and antioxidant protection, whereas the seed contributes emulsification, oil binding and structural stability, highlighting the synergistic technofunctional potential of the two fractions for low‐glycaemic, structured food products.

#### 3.4.4. FC and Stability

The foaming properties of flours depend generally on the surface tension formed by proteins, which keep air bubbles in suspension and slow the rate of coalescence [28]. In Table 3, TLP exhibited a moderate FC (23.63*%* ± 0.08*%*) and FS (11.89*%* ± 0.05*%*), reflecting the presence of soluble proteins and polyphenols capable of stabilizing air–water interfaces [10, 18]. Its fibrous microstructure supports the entrapment of air, contributing to foam formation. TSP showed a slightly lower FC (18.45*%* ± 0.02*%*), whereas FS (6.57*%* ± 0.11*%*) remained comparable. The reduced FC is attributable to the high lipid content, which can disrupt protein films at the interface, although sufficient proteins still maintain foam stability [26]. The FC and stability reported in this research were found to be within the ranges reported by Olawoye et al. [28] and Ijarotimi et al. [26] for different processed *A. viridis* seed flour (FC: 6.63%–36.03%; FS: 3.65%–26.66%) and groundnut seed flour (FC: 3.80%–6.32%), respectively, but lower than *Bougainvillea spectabilis* Willd. bracts powder (FC: 13.45%; FS: 57.72%) [34]. These findings indicate that TLP may be useful in aerated functional foods, whereas TSP provides moderate foaming that can complement composite formulations without compromising texture.

#### 3.4.5. LGC

The LGC, which is defined as the minimum flour concentration at which gel remains stable in an inverted tube, was used as an index of gelation capacity [28]. In Table 3, TLP formed gels at a lower concentration (LGC = 8.90*%* ± 0.42*%*) due to its high dietary fibre and polysaccharide content [11, 13], which promote water entrapment and network formation. A lower LGC reflects better gelation ability of the flour. This property is advantageous for low‐glycaemic gels, thickened porridges and structured snacks. Comparably, TSP required a slightly higher concentration (LGC = 11.71*%* ± 0.51*%*) for gelation, reflecting protein–lipid interactions that influence network formation. Despite these LGCs, the complementary gelation behaviours of TLP and TSP support their synergistic use in food formulations, where leaf contributes hydration and gel strength, and seed enhances texture and stability. With this, the resultant gels could provide viscoelastic matrices suitable for food systems such as bakery fillings, structured snack foods and pudding, which require thickening and gelling [28].

### 3.5. Flowability Properties

The bulk and tapped densities of TLP and TSP are presented in Table 4. TLP exhibited a low bulk density (0.42 ± 0.08 g/cm^3^) and tapped density of 0.57 ± 0.04 g/cm^3^, reflecting its porous and fibrous structure, discussed in Section 3.8.1. Such low‐density powders are advantageous for incorporating functional ingredients into composite flours, as they allow higher volume inclusion without excessive weight or compaction [28]. However, the high difference between tapped and bulk densities also contributes to higher compressibility [19], consistent with the elevated CI (26.53%) and HR (1.36) observed for TLP (Table 4). This behaviour is typical of fibre‐rich powders, which tend to interlock and trap air within irregular particles, leading to the closure of the airspace between the pores, reducing free‐flow properties [28, 35].

**Table 4:** Flow properties of *T. occidentalis* leaf and seed powders.

Parameters	TLP	TSP
Tap density (g/cm^3^)	0.57 ± 0.04^a^	0.64 ± 0.02^b^
Bulk density (g/cm^3^)	0.42 ± 0.08^a^	0.63 ± 0.02^b^
Carr′s index (%)	26.53 ± 0.01^b^	1.58 ± 0.04^a^
Hausner′s ratio	1.36 ± 0.02^b^	1.03 ± 0.04^a^
Angle of repose (°)	36.72 ± 0.41^b^	26.45 ± 0.22^a^

*Note:* Results are expressed as mean ± standard deviation (*n* = 3), and means with different superscripts in the same row are significantly (*p* < 0.05) different.

Abbreviations: TLP, *T. occidentalis* leaf powder; TSP, *T. occidentalis* seed powder.

In contrast, TSP exhibited a higher bulk density (0.63 ± 0.02 g/cm^3^) and tapped density (0.64 ± 0.02 g/cm^3^), reflecting its compact protein–lipid matrix. The smaller difference between bulk and tapped densities indicates lower compressibility and better packing, contributing to improved handling and flow properties [19]. This higher density is advantageous for food processing operations, such as dry blending, extrusion and packaging, where uniformity and controlled flow are critical [18]. Higher bulk density influences packing, dough handling and texture, and is desirable in applications such as bakery and extruded snacks, where structural integrity and uniform mixing are important [19].

The flowability parameters, expressed as CI, HR and angle of repose, are widely used to evaluate the handling, mixing and processing behaviour of powdered food ingredients [18]. In Table 3, TLP exhibited a higher CI (26.53%) and HR (1.36) compared with TSP, indicating moderate to poor flowability. The angle of repose of TLP (36.72°) was also higher, reflecting greater interparticle friction and cohesiveness. In contrast, TSP exhibited significantly lower CI (1.58%) and HR (1.03), together with a lower angle of repose (26.45°), indicating an excellent free‐flowing powder. The excellent flow behaviour of TSP can be attributed to its higher lipid content and more compact particle structure, which reduce interparticle friction and electrostatic interactions [13]. Seed flours such as amaranth [28], pumpkin seed, soybean and sesame have similarly been reported to show better flowability than fibrous leaf powders [8]. For instance, Olawoye et al. [28] reported that germinated amaranth flour had poor flowability (HR: 1.26, CI: 26.41%), which was greatly improved when the seeds were autoclaved (HR: 1.13, CI: 13.37%).

Although the relatively high CI and HR of TLP indicate poor powder flowability, which may adversely affect storage stability, hopper discharge and blending uniformity, these limitations may be mitigated in composite flour systems through incorporation with the more free‐flowing TSP or by applying particle engineering strategies such as agglomeration or the use of anticaking agents. Moreover, the reduced flowability of TLP is consistent with its SEM microstructure (in Section 3.8.1), which revealed a highly porous and fibrous network. Although this structure enhances water and glucose binding, it also increases surface area and mechanical interlocking between particles, leading to poorer flow [6]. Kumar et al. [34] reported similar CI (21.37%), HR (1.271) and angle of repose (32.09°) for *B. spectabilis* Willd. bracts powder. Koç et al. [19] reported CI and HR values between 19.64 and 32.58, and 1.29 and 1.49 for spinach. As observed in this research, this is typical of fibre‐rich plant powders, which tend to have irregular, porous and highly hygroscopic particles that promote particle–particle adhesion [18, 19].

In functional food applications, this suggests that TLP is best used in composite flours, premixes or hydrated systems rather than as a free‐flowing powder. The superior flowability of TSP makes it more suitable for dry blending, extrusion, tableting and packaging operations, whereas TLP contributes hydration, viscosity and functional bioactivity once incorporated into hydrated or semisolid food systems.

### 3.6. Colour Characteristics

The colour parameters (L*, a*, b*, hue angle, chroma and *Δ*E*) of TLP and TSP are presented in Table 5. Significant differences (*p* < 0.05) were observed between the two samples for all colour attributes, reflecting their distinct compositional profiles and pigment distribution.

**Table 5:** Colour characteristics of *T. occidentalis* leaf and seed powders.

Parameters	TLP	TSP
L*	37.68 ± 0.40^b^	71.32 ± 0.26^a^
a*	−7.45 ± 0.11^a^	3.42 ± 0.42^b^
b*	13.79 ± 0.20^b^	21.64 ± 0.42^a^
Hue angle (°)	118.38 ± 0.02^a^	81.02 ± 0.00^b^
Chroma (C*)	15.67 ± 0.04^b^	21.91 ± 0.06^a^
*Δ*E*	64.26 ± 0.00^a^	36.09 ± 0.01^b^

*Note:* Results are expressed as mean ± standard deviation (*n* = 3), and means with different superscripts in the same row are significantly (*p* < 0.05) different.

Asterisks (*) indicate standardised CIELAB colour system based on human vision.

Abbreviations: TLP, *T. occidentalis* leaf powder; TSP, *T. occidentalis* seed powder.

TLP exhibited lower lightness (L∗ = 37.68) and a negative a* value (−7.45), confirming its dark green appearance attributable to chlorophyll and polyphenol‐rich leaf tissues. In contrast, TSP showed significantly higher L* (71.32) and b* (21.64) values, indicating a lighter, yellowish flour typical of oilseed matrices containing carotenoids and lipid‐associated pigments [26]. The negative a* coordinate in TLP aligns with reports for polyphenol‐ and chlorophyll‐rich leafy matrices, where green pigments dominate colour expression [27]. Similar low L* and negative a* values have been reported in dehydrated leafy vegetable powders with high phenolic and chlorophyll contents [18, 19]. The relatively neutral to slightly positive a* value of TSP is consistent with the natural cream‐to‐light brown colour of pumpkin [4] and oilseed flours [26]. For instance, the natural colour of seed flour is beneficial for incorporation into bakery, beverage or composite flour formulations, where extreme pigmentation could affect consumer acceptability or sensory perception [26]. The higher hue angle of TLP (118.38°) confirmed dominance within the green region, whereas TSP (81.02°) shifted towards the yellow–red quadrant.

Chroma values were higher in TSP (21.91) than TLP (15.67), suggesting more saturated pigmentation, likely associated with carotenoid compounds and lipid‐soluble constituents [5, 31]. Furthermore, the substantial *Δ*E* difference between samples (> 30) indicates a clearly perceptible visual distinction, with implications for formulation and consumer acceptability [6]. Importantly, the darker green coloration of TLP correlated with its higher TPC and stronger antioxidant activity (DPPH, ABTS·^₊^ and FRAP), supporting previous reports that deeper green leafy matrices often exhibit elevated radical‐scavenging capacity due to chlorophyll–polyphenol synergy [7–9, 36]. In contrast, the lighter appearance of TSP aligned with its comparatively moderate antioxidant activity, reflecting lower phenolic density but contribution from lipid‐associated antioxidants such as tocopherols and carotenoids [10, 13].

From a functional perspective, the colour characteristics are consistent with the compositional findings of this study. The darker green tone of TLP reflects its high chlorophyll and polyphenol content, which are associated with strong antioxidant and enzyme‐inhibitory activities [10]. Meanwhile, the lighter, yellowish tone of TSP corresponds to its protein–lipid‐rich composition, supporting its technofunctional performance in emulsified and structured food systems [26]. Although TLP exhibited desirable functional and antiglycaemic properties, its relatively dark green colour may limit consumer acceptance in some food categories, particularly lightly coloured products. Consequently, its use may be better suited to products in which green pigmentation is acceptable or desirable, or at lower inclusion levels in composite formulations. Future studies should therefore evaluate sensory acceptability, colour stability during processing and storage and the performance of TLP and TSP in model food systems. Overall, the marked differences in colour parameters between TLP and TSP reinforce their complementary roles in functional food development, where both visual and bioactive properties must be considered to optimize product quality and consumer acceptance.

### 3.7. Antiglycaemic Properties

#### 3.7.1. *α*‐Amylase and *α*‐Glucosidase Inhibition

**Figure 3:**
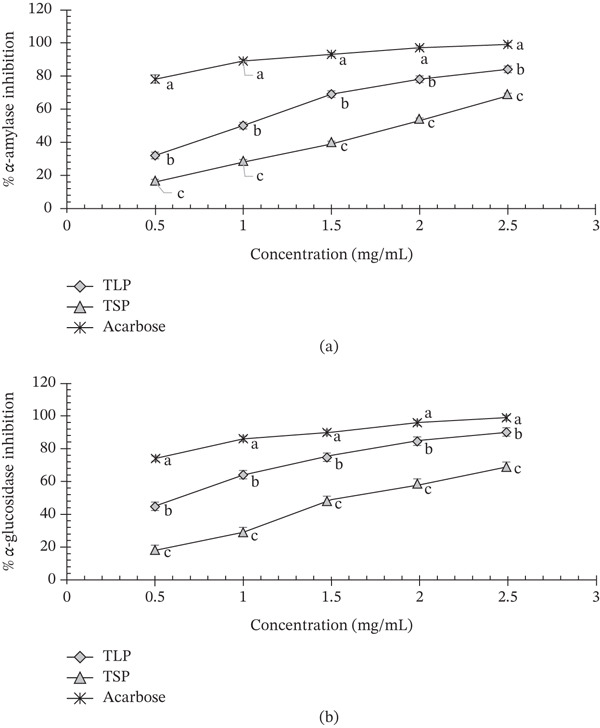
(a) *α*‐Amylase and (b) *α*‐glucosidase inhibitory activity of *T. occidentalis* leaf and seed powders. Values are mean ± SD (*n* = 3). Different superscript letters at the same time point indicate significant differences (*p* < 0.05) as determined by one‐way ANOVA followed by Tukey′s HSD test.

Figure 3 presents the inhibitory effects of TLP and TSP against *α*‐amylase and *α*‐glucosidase. TLP showed significantly (*p* < 0.05) stronger inhibition of both enzymes, approximately threefold than TSP, with IC_50_ values of 1.53 (*α*‐amylase) and 0.84 mg/mL (*α*‐glucosidase) (Table 6), reflecting its high polyphenol and flavonoid content (Table 2). Similar strong inhibitory activities have been reported for fluted pumpkin leaves [12, 16, 37] and other African leafy vegetables including *A. viridis* and *Solanum marcrocarpon* [8]. For instance, Oboh et al. [12] reported that unprocessed *T. occidentalis* had a significantly (*p* < 0.05) higher *α*‐amylase and *α*‐glucosidase inhibitory activities than blanched *T. occidentalis* at 0.17, 0.24, 0.14 and 0.18 mg/mL, respectively. Likewise, Oluwagunwa et al. [8] reported that *T. occidentalis* showed 100.00% *α*‐amylase inhibition at 1.8 mg/mL when compared with *S. marcrocarpon* (75.74%) and *A. viridis* (68.45%) at 2.3 mg/mL.

**Table 6:** The IC_50_ values of *T. occidentalis* leaf and seed powders against *α*‐amylase and *α*‐glucosidase enzymes.

Sample	*α*‐amylase IC_50_ (*μ*g/mL)	*α*‐glucosidase IC_50_ (*μ*g/mL)
TLP	1530 ± 0.08^b^	840 ± 0.02^b^
TSP	2580 ± 0.04^a^	2230 ± 0.00^a^
Acarbose	530 ± 0.05^c^	330 ± 0.02^c^

*Note:* Results are expressed as mean ± standard deviation (*n* = 3), and means with different superscripts in the same column are significantly (*p* < 0.05) different.

Comparatively, TSP exhibited moderate inhibition (IC_50_ = 2.58 mg/mL for *α*‐amylase and 2.23 mg/mL for *α*‐glucosidase), which can be attributed to its seed proteins, peptides and minor phenolics [10]. Working on *T. occidentalis* seed protein hydrolysates, Ruth et al. [10] reported similar concentration‐dependent *α*‐amylase and *α*‐glucosidase inhibitory activities, with IC_50_ values ranging from 1.03 to 1.31 mg/mL (acarbose: 0.33 mg/mL) and from 0.85 to 1.58 mg/mL, respectively. In vitro, the methanolic extract of *C. lanatus* seeds showed considerable inhibitory activity against *α*‐glucosidase with IC_50_ values of 54.44 *μ*g/mL and slight inhibitory activity against *α*‐amylase with IC_50_ values of 76.68 *μ*g/mL [27]. In addition, among prebiotics, Shirkhan et al. [5] reported that isomaltulose and pectin had antidiabetic activity by *α*‐amylase (IC_50_ = 11.36 mg/mL) and *α*‐glucosidase (IC_50_ = 2.38 mg/mL) inhibition, which is similar to the results of the current study. Oil‐rich seed flours such as sesame [35] and soybean [2] have similarly been reported to exert moderate inhibitory effects on carbohydrate‐digesting enzymes. Notably, soybean crude peptides had an inhibitory effect on porcine pancreatic *α*‐amylase and *α*‐glucosidase, and the inhibitory activity was proportional to the concentration of the soy proteins, with semi‐inhibitory concentrations of 18.795 ± 0.118 and 11.033 ± 0.033 mg/mL, respectively [2]. Although its polyphenol content was lower than that of TLP, the enzyme‐modulating effects of TSP may be enhanced by its protein–lipid matrix, which can interact with starch and digestive enzymes, limiting substrate accessibility [9, 10]. *α*‐Amylase hydrolyzes starch into dextrin, oligosaccharides and glucose. *α*‐Glucosidase is one of the key enzymes that decompose disaccharides into glucose. Inhibition of these enzymes can delay the hydrolysis of dietary starch in the digestive system, lower blood glucose levels, slow down glucose metabolism and delay glucose absorption [2].

One of the key therapeutic strategies for managing T2D is the control of hyperglycaemia through the inhibition of *α*‐amylase and *α*‐glucosidase, as these enzymes play central roles in postprandial glucose elevation [5]. In particular, *α*‐amylase is an important target for reducing blood glucose because it contributes significantly to glucose release, especially in noninsulin‐mediated diabetes mellitus and obesity‐associated hyperglycaemia [35]. When compared with acarbose, the *α*‐amylase IC_50_ of acarbose (0.53 ± 0.05 mg/mL) corresponds to 530 ± 50 *μ*g/mL, whereas the *α*‐glucosidase IC_50_ (0.33 ± 0.02 mg/mL) corresponds to 330 ± 20 *μ*g/mL. In comparison, TLP exhibited IC_50_ values of 1.53 ± 0.08 for *α*‐amylase and 0.84 ± 0.02 mg/mL for *α*‐glucosidase, which were significantly lower (*p* < 0.05) than those of TSP (2.58 ± 0.04 and 2.23 ± 0.00 mg/mL, respectively), indicating greater inhibitory potency of the leaf fraction but lower absolute inhibitory potency to acarbose (Table 6). Although acarbose exhibited significantly stronger inhibitory activity than both TLP and TSP (*p* < 0.05), the differences should be interpreted in the context of their fundamentally different modes of action. Acarbose is a purified pharmaceutical inhibitor that acts directly and specifically at the catalytic site of *α*‐amylase and *α*‐glucosidase, whereas TLP and TSP are complex whole‐food matrices whose inhibitory effects arise from the combined action of polyphenols, dietary fibre, proteins and other phytochemicals. Consequently, the higher IC_50_ values of TLP and TSP do not necessarily indicate poor efficacy but rather reflect the lower concentration of active inhibitory constituents within the crude powders. Shirkhan et al. [5] reported similar observations where acarbose had stronger *α*‐amylase (0.51 mg/mL) and *α*‐glucosidase (0.17 mg/mL) inhibitory activities than several prebiotics including inulin HP, FOS, isomaltulose and pectin. However, the inhibitory activities in this study were within the range reported in other natural plant studies including *T. occidentalis* seed protein hydrolysates [10], *T. occidentalis* leaves [12], soybean proteins [2] and New Zealand pine bark [9]. Importantly, unlike acarbose, which often causes gastrointestinal side effects [35], plant‐derived enzyme inhibitors offer both dietary and physiologically compatible strategies including glucose adsorption, delayed glucose diffusion and antioxidant protection [2]. Their *α*‐amylase cleaves *α*‐1,4 glycosidic bonds to convert complex dietary carbohydrates like starch into oligosaccharides and disaccharides, which are further broken down into absorbable monosaccharides such as glucose and fructose by glucosidases [5, 8]. Such multifunctional activity may be advantageous in dietary strategies for attenuating postprandial glycaemic response, while potentially avoiding the gastrointestinal side effects commonly associated with high dose acarbose therapy. As such, the comparison with acarbose is useful as a benchmark of relative potency but should not be interpreted as a direct equivalence between a pharmaceutical inhibitor and a functional food ingredient.

#### 3.7.2. Glucose Adsorption Capacity

**Figure 4:**
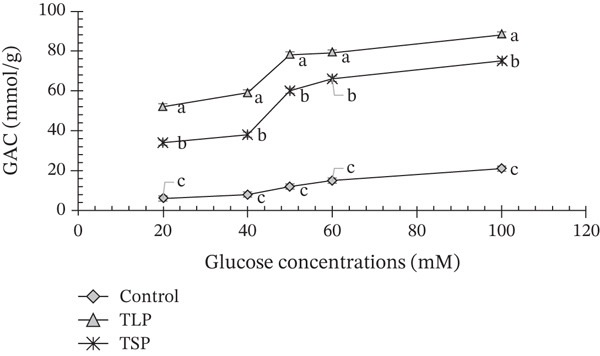
Glucose adsorption capacity (GAC) of *T. occidentalis* leaf powder (TLP) and seed powder (TSP) compared with control across glucose concentrations (10–100 mM). Values are mean ± SD (*n* = 3). Different superscript letters at the same time point indicate significant differences (*p* < 0.05) as determined by one‐way ANOVA followed by Tukey′s HSD test.

The glucose adsorption capacities of TLP and TSP increased progressively with increasing glucose concentration (20–100 mM), indicating concentration‐dependent binding behaviour (Figure 4). At all tested glucose concentrations, TLP exhibited significantly higher adsorption capacity than TSP (*p* < 0.05), whereas the control showed negligible binding. Similar glucose adsorption capacities have been reported for fruits [4, 27], leafy vegetables [6] and legumes [26] commonly used in glycaemic control formulations. This behaviour is typical of fibre‐rich plant materials, where polysaccharides and phenolic compounds provide binding sites for glucose molecules, reducing their diffusion and absorption in the intestinal lumen [27].

At 20‐mM glucose, TLP adsorbed approximately 50 mmol/g, increasing to nearly 85–90 mmol/g at 100 mM. In contrast, TSP demonstrated moderate adsorption, rising from approximately 30–35 mmol/g at 20 mM to 70–75 mmol/g at 100 mM. The higher adsorption capacity of TLP is consistent with its elevated TDF and porous microstructure, as observed by SEM. Dietary fibres, particularly soluble and hemicellulosic fractions, are known to entrap glucose molecules, increase matrix viscosity and reduce molecular mobility, thereby limiting glucose availability [18]. The substantial glucose‐binding ability of TLP may also be partially attributed to its polyphenol content. In addition, phenolic compounds can interact with carbohydrates via hydrogen bonding and noncovalent interactions, contributing to glucose retention within the matrix [10, 18]. This mechanism complements its previously observed *α*‐amylase and *α*‐glucosidase inhibition, suggesting a dual biochemical and physical modulation of carbohydrate availability.

Although TSP exhibited lower GAC than TLP, its adsorption capacity remained physiologically relevant. The moderate glucose binding observed for TSP likely arises from interactions between glucose and seed proteins [10, 35], as well as entrapment within the protein–lipid matrix visualized by SEM. Protein‐rich matrices have been reported to adsorb glucose through surface binding and matrix immobilization mechanisms, thereby reducing diffusion rates and postprandial glucose response [35]. Additionally, lipid components may contribute indirectly by influencing matrix hydrophobicity and mass transfer properties. The concentration‐dependent increase in GAC for both samples suggests that adsorption sites were not saturated within the tested range, supporting the presence of multiple binding domains associated with fibre, protein and phenolic constituents. When considered alongside glucose diffusion and enzyme inhibition results, the strong adsorption capacity of TLP and the moderate but sustained binding of TSP reinforce their complementary roles in attenuating glucose transport and moderating glycaemic response. Overall, the glucose adsorption behaviour confirms that *T. occidentalis* leaf and seed powders possess significant glucose‐binding potential, supporting their application as functional ingredients in low‐glycaemic food systems.

#### 3.7.3. Glucose Diffusion Behaviour

**Figure 5:**
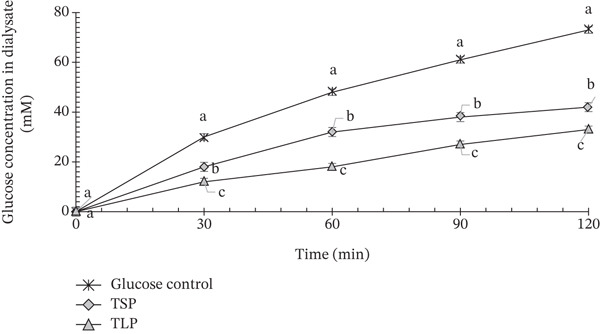
Effect of *T. occidentalis* leaf (TLP) and seed (TSP) powders on glucose diffusion across a dialysis membrane over 120 min. Values are mean ± SD (*n* = 3). Different superscript letters at the same time point indicate significant differences (*p* < 0.05) as determined by one‐way ANOVA followed by Tukey′s HSD test. Lower glucose levels indicate stronger glucose movement retardation.

The effect of TLP and TSP on glucose diffusion is shown in Figure 5. The glucose control exhibited rapid diffusion, with dialysate glucose increasing to approximately 70–75 mM within 120 min, representing unrestricted molecular transport typical of high‐glycaemic systems [14]. Both TLP and TSP significantly reduced glucose diffusion throughout the incubation period (*p* < 0.05). This effect is consistent with its high fibre and polyphenol contents, which inhibit *α*‐amylase and *α*‐glucosidase and physically entrap starch granules, limiting enzyme access [11, 12]. Similar diffusion‐retarding effects have been reported for fibre‐rich leafy vegetables [14], polyphenol‐dense plant matrices [10, 12] and legume seeds [4, 35], where glucose entrapment and viscosity effects delay molecular transport.

At 30 min, TLP limited glucose diffusion to approximately 10–15 mM compared with ~30 mM in the control, corresponding to an early‐stage reduction exceeding 50%. This retardation persisted over time, with glucose levels reaching only 30–35 mM at 120 min (~50% reduction). The strong diffusion‐limiting effect of TLP is consistent with its high dietary fibre content, porous microstructure and elevated water absorption capacity, which enhance glucose entrapment and increase medium viscosity [26, 35]. Fibre‐rich plant matrices have been widely reported to delay glucose transport across semipermeable membranes through physical restriction and reduced concentration gradients [10, 12, 27]. Additionally, polyphenols may contribute indirectly by interacting with carbohydrates or modifying matrix structure [27].

TSP also significantly slowed glucose diffusion, although to a lesser extent. Glucose levels increased gradually from 15 to 20 mM at 30 min to 40 to 45 mM at 120 min, representing a 30%–35% reduction relative to the control. The moderate but sustained retardation suggests a different structural mechanism, likely associated with protein‐mediated glucose adsorption and the formation of protein–lipid matrices that restrict molecular mobility [18, 21]. Oilseed flours and protein‐rich ingredients have similarly been shown to modulate carbohydrate diffusion through matrix entrapment and reduced mass transfer rates [26, 35]. The greater inhibitory effect observed for TLP aligns with its higher glucose adsorption capacity and fibre density, whereas TSP appears to exert its effect primarily through matrix structuring and lipid–protein interactions. When considered alongside *α*‐amylase, *α*‐glucosidase and lipase inhibition, these findings indicate that TLP and TSP regulate postprandial glycaemia through complementary physical and biochemical mechanisms. TLP predominantly limits glucose availability via fibre‐ and polyphenol‐mediated entrapment, whereas TSP provides structural modulation through its protein‐lipid network. Although the dialysis membrane assay provides a useful comparative measure of the ability of TLP and TSP to delay glucose movement, it does not fully replicate the physiological conditions of the small intestine, where mucus, unstirred water layers, epithelial transporters and peristaltic mixing influence glucose absorption. Consequently, the present results should be interpreted as indicative of relative glucose diffusion retardation rather than a direct measure of intestinal glucose uptake. Nonetheless, the glucose diffusion results support the potential of *T. occidentalis* leaf and seed powders as functional ingredients for attenuating glucose transport and moderating glycaemic response in composite food systems.

#### 3.7.4. Pancreatic Lipase Inhibitory Activity

**Figure 6:**
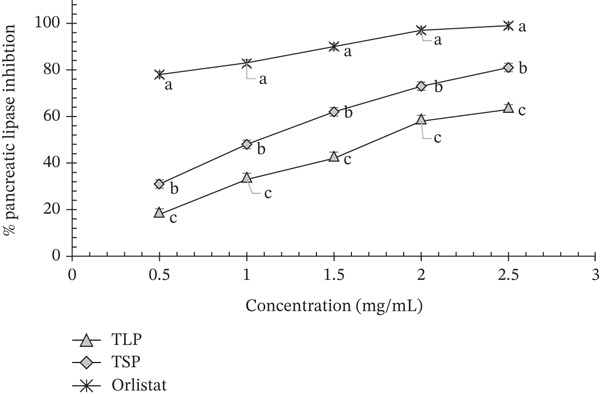
Pancreatic lipase inhibitory activity of TLP, TSP and orlistat at different concentrations (0.5–2.5 mg/mL). Values are mean ± SD (*n* = 3). Different superscript letters at the same time point indicate significant differences (*p* < 0.05) as determined by one‐way ANOVA followed by Tukey′s HSD test. Values represent the percentage inhibition of pancreatic lipase, showing a concentration‐dependent increase for all samples. TLP, *T. occidentalis* leaf powder; TSP, *T. occidentalis* seed powder.

The pancreatic lipase inhibitory activities of TLP, TSP and the reference inhibitor orlistat are presented in Figure 6, with corresponding IC_50_ values shown in Table 7. All samples exhibited a clear concentration‐dependent increase in inhibition (0.5–2.5 mg/mL), indicating dose‐responsive interaction with pancreatic lipase.

**Table 7:** Estimated IC_50_ values (mg/mL) for pancreatic lipase inhibition by *T. occidentalis* leaf powder (TLP), seed powder (TSP) and orlistat.

Sample	IC_50_ (*μ*g/mL)
TLP	1590 ± 0.02^a^
TSP	880 ± 0.06^b^
Orlistat	260 ± 0.02^c^

*Note:* Results are expressed as mean ± standard deviation (*n* = 3), and means with different superscripts in the same column are significantly (*p* < 0.05) different.

Orlistat, used as the positive control, showed the highest inhibitory activity across all concentrations, reaching approximately 95%–98% inhibition at 2.5 mg/mL, with a markedly low IC_50_ (260 ± 0.02 *μ*g/mL). This strong inhibition is consistent with its well‐established mechanism as a covalent inhibitor of pancreatic lipase, forming a stable acyl–enzyme complex that blocks triglyceride hydrolysis [8].

Among the *T. occidentalis* samples, TSP demonstrated significantly stronger lipase inhibition than TLP (*p* < 0.05) at all concentrations tested. At 2.5 mg/mL, TSP achieved approximately 78%–82% inhibition, compared with 60%–65% for TLP. The IC_50_ of TSP (880 ± 0.06 *μ*g/mL) was significantly lower than that of TLP (1590 ± 0.02 *μ*g/mL), confirming its higher potency. Although both powders were less active than orlistat, their inhibitory activities fall within the range reported for plant‐based lipase inhibitors derived from polyphenol‐rich leafy materials such as *A. viridis*, *Solanum macrocarpon* and *T. occidentalis* [8]. The stronger inhibition observed for TSP may be attributed to its full‐fat protein–lipid matrix and the presence of lipophilic bioactive compounds. Oilseed flours and protein‐rich plant materials have been reported to inhibit pancreatic lipase through multiple mechanisms, including interfacial competition, protein–enzyme interactions and the binding of hydrophobic phenolics to the active site or substrate interface [4, 35]. For instance, they can interact with lipase, while their lipid components may influence enzyme accessibility at the oil–water interface [35]. In addition, the high lipid content of TSP may facilitate the formation of substrate‐matrix complexes that reduce enzyme accessibility, whereas seed phenolics and peptides could contribute to direct enzyme inhibition. Comparable inhibitory patterns have been observed in oilseed flours and protein‐rich plant matrices such as pumpkin seed, soybean and other cucurbit seed extracts, where bioactivity arises from synergistic interactions among phenolics, peptides and lipophilic constituents [4, 35, 38].

In contrast, the moderate inhibition exhibited by TLP likely reflects its different compositional profile, characterised by higher dietary fibre and polyphenol content but lower lipid levels. Polyphenols are known to inhibit pancreatic lipase by forming noncovalent complexes with the enzyme and altering its conformation [5, 8]. Additionally, soluble fibre can increase viscosity and limit enzyme–substrate interactions at the oil–water interface [26]. However, the absence of a lipid‐rich matrix may explain the comparatively higher IC_50_ of TLP relative to TSP. Similar lipase inhibition has been reported for polyphenol‐rich leafy vegetables and plant extracts used in metabolic health studies [8, 12, 24].

From a functional perspective, the lipase inhibitory activities of both TLP and TSP suggest potential to modulate postprandial lipaemia alongside glycaemic responses. Pancreatic lipase inhibition contributes to reduced lipid hydrolysis and slower fatty acid release, mechanisms associated with improved metabolic regulation and reduced postprandial lipaemia [1, 3]. Although their potency does not match that of orlistat, plant‐derived inhibitors are generally associated with milder effects and fewer gastrointestinal side effects, making them attractive for incorporation into functional foods targeting metabolic syndrome and T2D [1, 6]. The combined inhibition of *α*‐amylase, *α*‐glucosidase, glucose diffusion and pancreatic lipase observed in this study indicates a multitarget metabolic modulation strategy. Overall, these findings demonstrate that *T. occidentalis* powders, particularly the full‐fat seed powder, possess physiologically relevant pancreatic lipase inhibitory activity. When integrated into composite food systems, they may contribute to attenuated fat digestion and improved metabolic control through complementary biochemical and physical mechanisms.

### 3.8. Microstructural Characteristics

#### 3.8.1. SEM Micrographs

**Figure 7:**
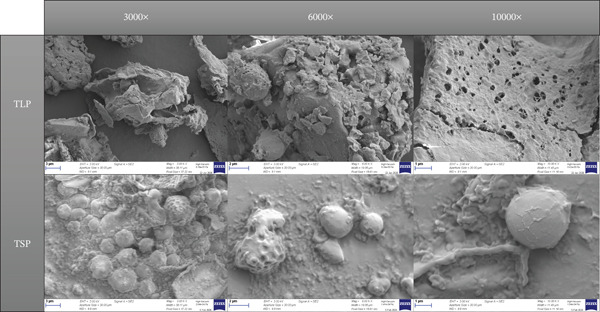
SEM micrographs of *T. occidentalis* leaf powder (TLP) and *T. occidentalis* seed powder (TSP) at 3000×, 6000× and 10000× magnification.

The SEM micrographs of TLP and TSP revealed distinct microstructural features that explain their contrasting technofunctional and antiglycaemic properties (Figure 7).

TLP exhibited an irregular, fragmented structure with thin, plate‐like particles, rough surfaces and extensive porosity. Numerous voids, fissures and interconnected pores were evident, particularly in the higher magnification images, indicating disruption of the plant cell wall matrix during drying and milling. This highly porous architecture is characteristic of fibre‐rich leafy materials and has been widely reported for plant powders with high dietary fibre and polysaccharide content rich in cellulose, hemicellulose and pectic substances such as spinach [19] and African nightshade [31], where the high surface area facilitates water penetration and solute binding.

The presence of open pores and fractured cell wall remnants increases the available surface area for water penetration and solute interaction, providing a structural basis for the high water absorption capacity, glucose adsorption, and diffusion‐retarding effects observed for TLP. In addition, the rough, irregular surfaces observed in TLP may facilitate the adsorption of digestive enzymes and glucose molecules, contributing to the strong *α*‐amylase and *α*‐glucosidase inhibition reported in this study. The fragmented nature of TLP particles also explains its low bulk density and poor flowability, as irregular shapes and high interparticle friction hinder efficient packing. Such microstructural characteristics are typical of dried leafy powders [18, 19] and are consistent with the elevated CI and angle of repose (Table 4) observed in the present study. Similar porous microstructures have been linked to enhanced glucose‐binding capacity and delayed glucose transport in fibre‐rich plant powders such as *Moringa oleifera* and *Amaranthus* spp. [6, 23].

In contrast, TSP displayed a more compact and aggregated structure, dominated by spherical and oval granules embedded within a continuous matrix. Several micrographs revealed smooth, rounded oil bodies or lipid droplets, often surrounded by proteinaceous material, forming a characteristic protein–lipid matrix. This morphology is typical of full‐fat oilseed flours and has been reported for peanut [26, 33] and pumpkin seeds [4], containing intact lipid bodies. The relatively smooth surfaces and spherical particles observed in TSP promote closer packing and reduced interparticle friction, explaining its higher bulk and tapped densities, lower CI and improved flowability compared with TLP. The presence of intact lipid bodies also accounts for the high oil absorption capacity and superior emulsifying activity and stability, as proteins surrounding oil droplets can readily adsorb at oil–water interfaces and stabilise emulsions [26, 33].

From a physiological perspective, the compact protein–lipid network observed in TSP is likely to restrict the diffusion of glucose and digestive enzymes, thereby slowing carbohydrate hydrolysis and glucose release. Similar microstructures have been associated with reduced starch digestibility and delayed glucose diffusion in oilseed‐based and protein‐rich food matrices [26, 35]. This structural feature provides a mechanistic explanation for the moderate but sustained glucose diffusion retardation and enzyme inhibition exhibited by TSP in the present study. Overall, the SEM analysis highlights clear structure‐function relationships between TLP and TSP. The highly porous, fibrous microstructure of TLP underpins its strong water binding, glucose adsorption, antioxidant activity and enzyme inhibition, making it particularly effective for glycaemic regulation. Conversely, the compact, protein–lipid‐dominated microstructure of TSP supports its excellent emulsifying and foaming properties, improved flowability and moderated glucose diffusion. These complementary microstructural features suggest that combining TLP and TSP in composite formulations could yield functional foods with balanced processing performance and enhanced metabolic benefits.

#### 3.8.2. FTIR Spectral Analysis

**Figure 8:**
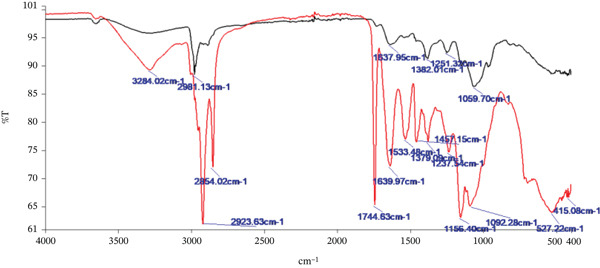
FTIR spectra of TLP (black) and TSP (red) with labelled characteristic bands. TLP, *T. occidentalis* leaf powder; TSP, *T. occidentalis* seed powder.

The FTIR spectra of TLP and TSP are presented in Figure 8. Both materials exhibited complex spectra reflecting their heterogeneous biochemical compositions; however, distinct differences were observed that are consistent with their contrasting nutritional and functional profiles. The spectrum of TLP was dominated by a broad and intense absorption band in the region of 3200–3400 cm^−1^, corresponding to O–H stretching vibrations associated with hydroxyl groups of polyphenols, cellulose, hemicellulose and pectic polysaccharides [18, 31]. This strong O–H signal is a characteristic of plant materials rich in dietary fibre and phenolic compounds, and supports the high TPC, antioxidant activity and water absorption capacity observed for TLP in this study. Additional absorption bands around 1600–1700 cm^−1^ were attributed to C=O stretching of aromatic and phenolic compounds, further confirming the abundance of bioactive phenolics in the leaf [18]. These functional groups are directly linked to the biological activity of TLP. Hydroxyl and carbonyl groups enable polyphenols to donate hydrogen atoms or electrons, explaining the strong DPPH, ABTS and FRAP activities (Table 2), while also facilitating interactions with digestive enzymes such as *α*‐amylase and *α*‐glucosidase, leading to enzyme inhibition (Figure 3) and contributing to reduced glucose diffusion and enhanced glucose adsorption capacity (Figure 4).

In contrast, the FTIR spectrum of TSP exhibited prominent bands in the regions of 2800–3000 cm^−1^, corresponding to C–H stretching vibrations of aliphatic chains and strong absorptions near 1740 cm^−1^, which are characteristic of ester carbonyl (C=O) groups of lipids and triglycerides [18]. These features confirm the high lipid content of the full‐fat seed flours. In addition, distinct peaks around 1540–1650 cm^−1^ (Amides I and II bands) and 3200–3400 cm^−1^ (N–H stretching) were observed, reflecting the presence of seed storage proteins [17, 26]. The coexistence of protein and lipid functional groups in TSP explains its strong emulsifying capacity, oil holding and gelation behaviour (Table 3). Protein molecules provide surface‐active sites for stabilizing oil–water interfaces, whereas lipid functional groups contribute to hydrophobic interactions and flavour retention in food systems [17]. Furthermore, the protein–lipid matrix indicated by the FTIR spectrum supports the observed reduction in glucose diffusion (Figure 4) and enhanced glucose adsorption capacity (Figure 3), as such matrices can physically restrict glucose mobility and modulate enzyme accessibility, thereby limiting glucose availability during in vitro digestion [26, 35].

**Figure 9:**
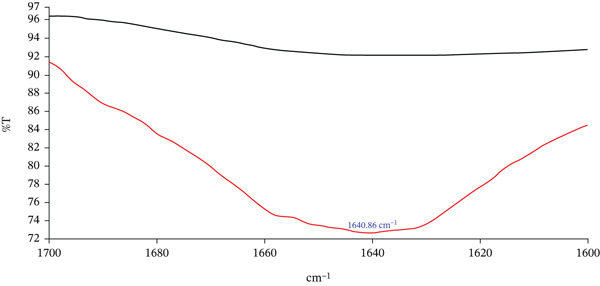
FTIR of *T. occidentalis* leaf powder (TLP, black line) and seed powder (TSP, red line) in the Amide I region (1700–1600 cm^−1^). The spectra highlight differences in protein structural features, with TSP exhibiting a broader and more intense absorption band centred around ~1640 cm^−1^ compared with TLP.

Furthermore, the Amide I region (1700–1600 cm^−1^) of the FTIR spectra, which is primarily associated with C=O stretching vibrations of peptide bonds and is sensitive to protein secondary structure, was further examined to explore structural differences between TLP and TSP (Figure 9). Both samples exhibited broad and overlapping absorption bands within this region, reflecting the complex and heterogeneous nature of plant protein matrices. TLP showed a relatively broadened band with a slight shift towards lower wavenumbers compared with TSP, suggesting a higher contribution of disordered or aggregated structures. Such band broadening and shifts are commonly associated with increased hydrogen bonding and structural heterogeneity, which may arise from interactions between proteins and polyphenolic compounds. In contrast, TSP displayed a comparatively sharper and more defined Amide I band, indicating a more ordered protein conformation, likely associated with its higher protein and lipid content and reduced interference from phenolic constituents. Attempts to resolve the Amide I band into individual secondary structural components (*α*‐helix, *β*‐sheet, *β*‐turn and random coil) using deconvolution and Gaussian fitting were limited by significant peak overlap and low spectral resolution of individual subbands. Similar limitations of Amide I deconvolution in heterogeneous plant protein systems have been noted previously [37, 39], where broad overlapping bands can compromise the reliability of secondary‐structure fitting. Nevertheless, the observed differences in band position and profile between TLP and TSP provide qualitative evidence of structural variation, potentially reflecting noncovalent interactions such as hydrogen bonding between phenolic hydroxyl groups and protein backbone amide groups.

Overall, although the FTIR analysis of the Amide I region does not allow definitive quantification of secondary structure, it indicates molecular‐level evidence for their contrasting but complementary functional roles. The polyphenol‐ and fibre‐rich chemical signature of TLP underpins its antioxidant and enzyme inhibitory activities, whereas the protein‐ and lipid‐dominated spectrum of TSP supports its technofunctional performance and digestion‐modulating effects. These structure‐function relationships reinforce the suitability of *T. occidentalis* leaf and seed flours as integrated ingredients for the development of low‐glycaemic, antioxidant‐rich functional foods.

## 4. Conclusions

This study demonstrates that *T. occidentalis* leaf (TLP) and seed (TSP) powders exhibit complementary physicochemical, technofunctional and metabolic properties that support their application in diabetes‐oriented functional foods. TLP, characterised by high dietary fibre and polyphenol contents, showed strong antioxidant activity, elevated water and glucose adsorption capacities, significant glucose diffusion retardation and potent *α*‐amylase and *α*‐glucosidase inhibition. In contrast, the full‐fat TSP, rich in protein and lipids, displayed superior emulsifying, oil‐holding, foaming and gelation properties, alongside moderate antiglycaemic and notable pancreatic lipase inhibitory activity. Collectively, these attributes indicate multitarget modulation of postprandial glycaemia and lipid digestion through both biochemical (enzyme inhibition) and physical (glucose entrapment and diffusion control) mechanisms. The complementary functionality of TLP and TSP highlights their potential for incorporation into composite, plant‐based formulations designed to attenuate postprandial hyperglycaemia while maintaining desirable processing performance. Nevertheless, the absence of complementary structural analyses such as SDS‐PAGE, fluorescence spectroscopy or specific binding assays limits direct confirmation of polyphenol–protein interactions, and these techniques should be incorporated in future studies to strengthen mechanistic understanding. In addition, in vivo validation through animal and human intervention studies is necessary to substantiate these in vitro findings. Further animal and/or human intervention studies are required to confirm the physiological relevance of the present in vitro findings. In particular, investigation of potential modulation of intestinal glucose transporters such as GLUT2, together with evaluation of longer term metabolic effects, would provide deeper mechanistic insight and strengthen the translational relevance of these results.

## Author Contributions

**Mary Nkongho Tanyitiku:** conceptualization, methodology, investigation, data curation, formal analysis, funding acquisition, writing—original draft, and writing—review & editing. **Igor Casimir Njombissie Petcheu:** conceptualization, methodology, investigation, writing—original draft, and writing—review & editing.

## Funding

No funding was received for this manuscript.

## Conflicts of Interest

The authors declare no conflicts of interest.

## Data Availability

The data that support the findings of this study are available from the corresponding author upon reasonable request.
